# Evaluating the Significance of Viscoelasticity in Diagnosing Early-Stage Liver Fibrosis with Transient Elastography

**DOI:** 10.1371/journal.pone.0170073

**Published:** 2017-01-20

**Authors:** Jingxin Zhao, Fei Zhai, Jun Cheng, Qiong He, Jianwen Luo, Xueping Yang, Jinhua Shao, Huichun Xing

**Affiliations:** 1 Beijing Ditan Hospital, Capital Medical University, Beijing, China; 2 Hisky Medical Technology Co., Ltd, Beijing, China; 3 Department of Biomedical Engineering, Tsinghua University, Beijing, China; Kaohsiung Medical University, TAIWAN

## Abstract

Transient elastography quantifies the propagation of a mechanically generated shear wave within a soft tissue, which can be used to characterize the elasticity and viscosity parameters of the tissue. The aim of our study was to combine numerical simulation and clinical assessment to define a viscoelastic index of liver tissue to improve the quality of early diagnosis of liver fibrosis. This is clinically relevant, as early fibrosis is reversible. We developed an idealized two-dimensional axisymmetric finite element model of the liver to evaluate the effects of different viscoelastic values on the propagation characteristics of the shear wave. The diagnostic value of the identified viscoelastic index was verified against the clinical data of 99 patients who had undergone biopsy and routine blood tests for staging of liver disease resulting from chronic hepatitis B infection. Liver stiffness measurement (LSM) and the shear wave attenuation fitting coefficient (AFC) were calculated from the ultrasound data obtained by performing transient elastography. Receiver operating curve analysis was used to evaluate the reliability and diagnostic accuracy of LSM and AFC. Compared to LSM, the AFC provided a higher diagnostic accuracy to differentiate early stages of liver fibrosis, namely F1 and F2 stages, with an overall specificity of 81.48%, sensitivity of 83.33% and diagnostic accuracy of 81.82%. AFC was influenced by the level of LSM, ALT. However, there are no correlation between AFC and Age, BMI, TBIL or DBIL. Quantification of the viscoelasticity of liver tissue provides reliable measurement to identify and differentiate early stages of liver fibrosis.

## Introduction

Liver fibrosis is generally caused by an excess accumulation of extracellular matrix proteins and occurs in most types of chronic liver diseases. The etiology of liver fibrosis is multifactorial in nature, with viral infection, alcohol overuse, and nonalcoholic steatohepatitis being common causes [1]. Liver fibrosis is an important health issue as it generally leads to liver cirrhosis and even cancer and, therefore, it is associated with serious long-term consequences in terms of patient morbidity and mortality [2].

A process of liver fibrosis is identifiable in most chronic liver diseases. As liver fibrosis in its early stages can be reversed, early prompt and accurate diagnosis is clinically important. Diagnosis of liver fibrosis by biopsy is widely used in clinical practice and is considered as the gold standard for diagnosis [3]. However, liver biopsy is a relatively expensive and invasive procedure associated with patient discomfort and a small risk of serious bleeding. Moreover, biopsy requires specialist histological examination for accurate staging of the liver disease. As conventional medical imaging techniques (e.g., X-ray, computed tomography, magnetic resonance imaging, and ultrasound imaging) do not provide sufficient quantitative information to diagnose liver fibrosis, biopsy continues to be used despite its invasive nature and the aversive feelings it provokes in many patients [4,5].

Over the last decade, novel non-invasive diagnostic techniques, based on ultrasound imaging, have been developed to provide an assessment of the degree of fibrosis and cirrhosis from measurement of the stiffness of liver tissue [6]. Among these techniques, transient elastography (TE), performed using Fibroscan (Echosens, Paris) [7] or FibroTouch (Hisky Co., Ltd, Wuxi) [8,9], has been the most widely used evaluation method. TE is non-invasive and provides measures of liver elasticity that are highly accurate and reliable for the identification of liver fibrosis and cirrhosis [10,11]. In recent years, TE technology has greatly advanced and has become the most useful clinical test for assessing moderate to high stages of liver fibrosis. TE is valuable for the diagnosis of moderate liver fibrosis and much higher stage, especially for the identification of severe liver fibrosis and early cirrhosis. However, TE is not reliable for the diagnosis of mild liver fibrosis, and especially for differentiating early F1 and F2 stages of liver fibrosis [12–14].

The liver is a viscoelastic structure. An increase in viscosity leads to an increase in energy loss (i.e., relative motions within the liver tissue decrease as viscosity increases). Change in viscosity is closely associated with the status of liver disease [15]. Although viscosity is an important property of the health status of liver tissue, clinically, it has largely been ignored. Only recently has the practical value of viscosity for the early diagnosis of liver disease been considered [15]. Salameh and Larrat [16] demonstrated that changes in viscosity were associated with fatty degeneration, indicative of early stages of liver fibrosis, while elasticity values remained within normal limits of healthy liver tissue, with no observable evidence of liver fibrosis. The viscosity properties of the liver can therefore facilitate early identification of liver fibrosis. Not considering measurement of liver viscosity could result in a missed diagnosis of liver fibrosis [17].

Recent studies evaluating the viscosity of liver tissue have reported variation in the velocity of transmission of mechanical vibration of different frequencies within viscoelastic materials [18]. In a research setting, sinusoidal mechanical vibration at different frequencies can be applied to the surface of a tissue and the corresponding shear wave velocity within the tissue can be computed simultaneously [19]. However, in clinical practice, current systems of TE do not allow the use of different frequencies of vibration, which is a requirement for accurate quantification of tissue viscoelasticity.

The aim of our study was to establish a new viscoelasticity parameter for TE, based on the mechanics of viscoelasticity, using finite numerical simulations in combination with clinical trial for verification. This new index could assist clinicians in accurate diagnosis of liver fibrosis in early stage.

## Materials and Methods

### Mathematical model

A viscoelastic body, such as the liver, can be described by a spring and damper model. Under alternating stress deformation, viscoelastic materials will both store energy and dissipate energy as heat. There are several models of viscoelastic materials that can be used to model the liver. In this study, we used the viscoelastic properties of Kelvin-Voigt and Maxwell materials for this purpose.

A Kelvin-Voigt material, also called a Voigt material, is a common viscoelastic material having both elasticity and viscosity properties. As the liver is a viscoelastic structure, characterization of both its tissue elasticity (stiffness) and its viscosity would have important medical applications as each of these properties is closely related to pathological changes in the liver [17]. The general linear viscoelastic model is expressed as:

$$\left(a_{ijkh}^{\left(0\right)}+a_{ijkh}^{\left(1\right)}\frac{\partial }{\partial t}+\cdots +a_{ijkh}^{\left(m\right)}\frac{\partial^{m}}{\partial t^{m}}\right)\sigma_{kh}=(b_{ijkh}^{\left(0\right)}+b_{ijkh}^{\left(1\right)}\frac{\partial }{\partial t}+\cdots +b_{ijkh}^{\left(n\right)}\frac{\partial^{n}}{\partial t^{n}})\varepsilon_{kh}$$ (1)

where *σ* defines the stress of the tissue and *ε* its strain. This general formula explains the functional relationship between the strain and stress of the material over time. The material’s elastic and viscosity parameters ($a_{ijkh}^{\left(l\right)}(l=0,\ldots ,m)$ and $b_{ijkh}^{\left(l\right)}(l=0,\ldots ,n)$) can be determined by solving Eq (1) for *m* and *n*.

For an isotropic viscoelastic body, assuming that tensile properties of the tissue are expressed along only one dimension, Eq (1) can be re-arranged as,

$$\left(a_{}^{\left(0\right)}+a_{}^{\left(1\right)}\frac{\partial }{\partial t}+\cdots +a_{}^{\left(m\right)}\frac{\partial^{m}}{\partial t^{m}}\right)\sigma =(b_{}^{\left(0\right)}+b_{}^{\left(1\right)}\frac{\partial }{\partial t}+\cdots +b_{}^{\left(n\right)}\frac{\partial^{n}}{\partial t^{n}})\varepsilon$$ (2)

where *m* = 0 and *n* = 1 to describe the viscoelastic properties of a Voigt material.

In clinical practice, the stiffness of liver tissue, or its Young’s modulus, can be directly measured using the commercially available FibroScan or FibroTouch systems. This stiffness value is widely used as an auxiliary measurement for the diagnosis of liver fibrosis. Under conditions of isotropic elasticity, the elasticity modulus *E* of the tissue can be calculated as:

$$E=2\times \mu_{1}\times \left(1+\sigma \right)$$ (3)

where *μ*_1_ is the tissue shear elasticity and σ is the Poisson's ratio. As liver tissue is virtually incompressible, Poisson's ratio was set to be 0.4999. In our simulations, we applied viscosity, elasticity and other parameters to our two-dimensional model, with the displacement data, in the transient analysis, calculated by applying Eqs (2) and (3). We evaluated the effect of viscosity on the characteristics of shear wave propagation by processing the displacement data. Numerical simulations were conducted for five viscosity and four elasticity values: viscosity modulus (*μ*_2_) values of 0, 0.5, 1, 2, and 4 Pa·s; and elasticity modulus (*E*) values of 4, 6, 8 and 10 kPa [17–19].

### Finite element model

A TE probe was used to generate sinusoidal mechanical vibrations, oriented perpendicular to the liver tissue to induce a shear wave. As the shear wave propagates through the tissue, the ultrasound transducer, which is tightly integrated with the mechanical vibration probe, records ultrasound radiofrequency data along the scanning line. Information on the distribution of the shear wave through the material is contained in the data. Therefore, processing of the radiofrequency data provides the axial displacement distribution of the tissue at different depths as a function of time of propagation of the wave. We used the axial displacement data to evaluate the effect of varying viscoelastic parameters on the characteristics of shear wave propagation.

**Fig 1:**
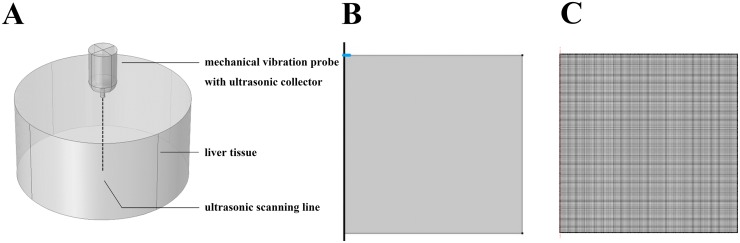
Finite element model of liver tissue and the mesh generated. (A) An idealized 3D model. (B) An idealized two-dimensional axisymmetric finite element model of liver tissue adopted in our study. (C) The generated finite element mesh (22,500 finite elements).

As shown in Fig 1A, an axisymmetric model was used for numerical simulations. It includes the TE probe and liver tissue. An idealized two-dimensional axisymmetric finite element model was built to simulate shear wave propagation within liver tissue. COMSOL Multiphysics (Stockholm, Sweden) and MATLAB (Natick, MA, USA) were used for model construction and shear wave simulation. Testing of the model indicated that results from our two-dimensional asymmetric model approximated those of a three-dimensional model under the same condition (Fig 1A). Therefore, we deemed our two-dimensional model was valid to investigate the viscoelastic properties of liver tissue, providing higher quality of the measurement grid, better convergence and much shorter computational time than a three-dimensional model. It is important to note that the higher the quality of the measurement grid, the more reliable and accurate the results of the numerical simulation.

As shown in Fig 1B, a 15 cm×15 cm model size was used to represent the liver, with the thick line on the left side representing the symmetry axis. In three-dimensional space, the model was represented as an axisymmetric cylinder. A probe with a 0.008 m diameter was used. The parameters of the mechanical vibration were as follows: frequency, 50 Hz; period, 1; and amplitude, 1 mm. As the ultrasound transducer is tightly coupled to the probe in the TE systems, ultrasound radiofrequency data along the scanning line are collected from the transducer during shear wave propagation.

The effect of varying tissue viscosity on the characteristics of shear wave propagation was evaluated using finite element analysis. Simulation time was set as 0.15 s, which was the time needed for the shear wave to propagate to the boundary of the model. A time step for analysis of 5.0×10^−5^ s was used, this shorter interval providing more accurate results than larger intervals. As the size and shape of the mesh affects the accuracy of finite element analysis, we generated a square-shaped mesh (best schemes of finite element meshing, elements quality average value, 1.0; element area ratio, 1) containing 22,500 finite elements (Fig 1C). This mesh was sufficient to fully quantify our idealized two-dimensional liver model.

### Clinical data

This study was approved by the ethical committee from Beijing Ditan Hospital and study protocol was in accordance with the Declaration of Helsinki. Data was collected from the medical records and stored in a locked study member’s computer without patients’ identifiers. All participants provided their written informed consent before they participated in this study. Participant consents were reserved in hospital case. The consent procedure was approved by Ethics Committee of Beijing Ditan Hospital Capital Medical University.

The patients’ data ware acquired between April 2014 and February 2015 from Beijing Ditan Hospital Capital Medical University. We completed TE assessments, for staging of liver fibrosis, in 108 patients with chronic hepatitis B, according to the 2010 guidelines for the prevention and treatment of chronic hepatitis B [20]. All patients underwent liver biopsy (LB), with METAVIR scoring of the stage of liver fibrosis, and routine blood tests (BT) obtained on the same day as the TE assessment [21,22]. All patients were not received antiviral therapy treatment. All patients provided informed consent prior to biopsy. All participants provide their written informed consent to participate in this study. Participant consents were reserved in hospital case. The consent procedure was approved by Ethics Committee of Beijing Ditan Hospital Capital Medical University.

Exclusion criteria: history of antiviral therapy; concurrent infection with other viruses; decompensated cirrhosis; hepatocellular carcinoma; hepatic failure, and other liver diseases; any malignancy during the study period; liver biopsy specimen smaller than 15 mm; failure of measurement of LSM; alcohol ingestion in excess of 40 g/day for more than 5 years; and serum ALT concentrations more than five times the upper limit of normal (42 IU/L in both sexes); pregnant women. Liver fibrosis stage >F2 according to liver biopsy. the size of the biopsy sample <15mm.

LSM values were obtained directly using the FibroTouch system. The assessment of LSM was performed by an experienced operator who was blinded to patients’ clinical and laboratory data. For the assessment, patients were placed in a standardized supine position, with the right hand placed under their head. The coupling agent was smeared over the region of the right 7^th^-9^th^ intercostal spaces. The TE probe was applied to the skin, in a vertical position, with pressure maintained within the range permitted scope. LSM values were subsequently expressed in kilopascals (kPa). LSM values were obtained until the success rate (number of valid acquisitions divided by number of attempts) was >60% and the ratio of interquartile range of LMS values to the median value of measures was <30%. Overall, a median value of 10 successful measurements was considered as valid. The shear wave attenuation fitting coefficient (AFC) was then calculated by processing the ultrasound radiofrequency data, as previously described.

### Liver biopsy

Ultrasound-guided percutaneous liver biopsy was performed using a 16 G disposable needle (Hepafix, B. Braun, Melsungen, Germany) under local anesthesia immediately following the LSM assessment. Specimens of a minimum length of 15 mm were immediately fixed in 10% formalin for further analysis. All biopsy samples were reviewed independently by two histopathologists who were blinded to the clinical data. Liver fibrosis was classified into five stages using the METAVIR scoring system, as follows [23]: F0, no fibrosis; F1, mild fibrosis with no identifiable fibrous septum; F2, fibrosis with a few fibrous septa; F3, numerous septa present but with no evidence of cirrhosis; and F4, cirrhosis. Significant liver fibrosis was defined as F2 or greater (F ≥2).

### Statistical analysis

All data were expressed as the mean±standard deviation. Comparative analysis of the LSM and AFC was performed using a single factor analysis of variance (LSD) and independent-samples *t*-test, as appropriate for the data type. Spearman’s correlation coefficient was calculated between fibrosis stage and the LSM/AFC. Decision on the presence or absence of hepatic fibrosis was set by the LSM cut off (≥F1). The diagnostic parameters of the LSM and AFC were evaluated using receiver operating characteristic (ROC) curve analysis on all available patients for each test. Significant differences between ROC curves were evaluated using the method proposed by Delong [24] for paired patient data. The 95% confidence intervals of mean estimates were calculated using the bootstrap method [25]. For all analyzes, *P <0.05, **P <0.01 and *** P <0.001 were considered to be statistically significant, as appropriate for the dataset. Optimal cut-offs were identified by maximizing Youden’s index as follows: J = Sensitivity + Specificity—1. Moreover, sensitivity, specificity, positive predictive value (PPV), negative predictive value (NPV) and diagnostic accuracy were calculated for the LSM and AFC. All analyzes were performed using IBM SPSS Statistics version 19.0 (SPSS Inc., USA).

## Results

### Numerical simulations

#### Axial displacement in scanning line

**Fig 2:**
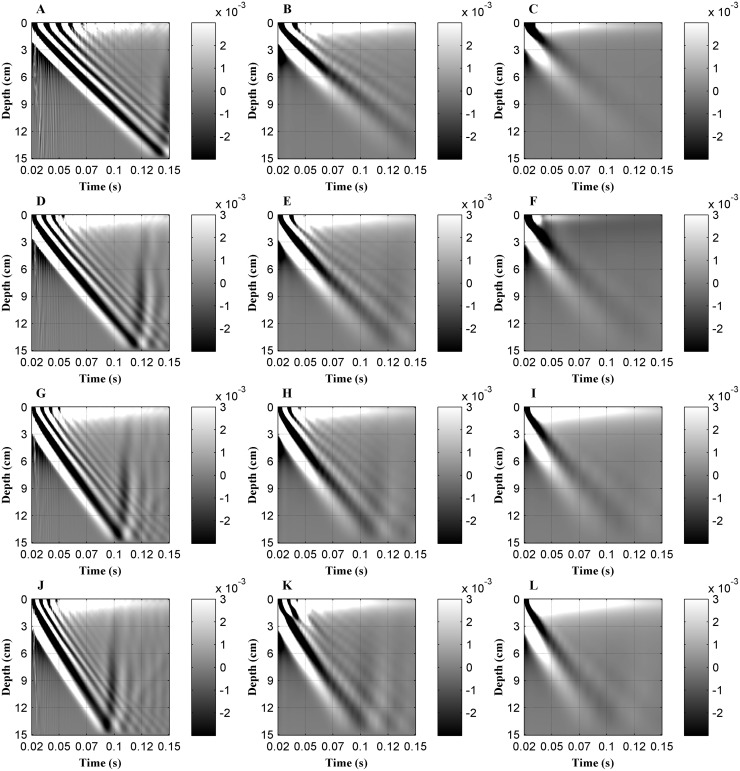
Axial displacement, produced by an external mechanical vibration, measured along the scanning line in the liver tissue model as a function of time (0.02s to 0.15s after excitation). **The following combinations of Young's modulus (*E*) and viscosity (*μ***_**2**_**) were used for simulations:** (A) 4 kPa+0 Pa·s, (B) 4 kPa +1 Pa·s, (C) 4 kPa+4 Pa·s, (D) 6 kPa+0 Pa·s, (E) 6 kPa+1 Pa·s, (F) 6 kPa+4 Pa·s, (G) 8 kPa+0 Pa·s, (H) 8 kPa+1 Pa·s, (I) 8 kPa+4 Pa·s, (J) 10 kPa+0 Pa·s, (K) 10 kPa+1 Pa·s and (L) 10 kPa+4 Pa·s, respectively.

Fig 2 shows the axial displacement as a function of time (0.02 s to 0.15 s after excitation). A dynamic range of displacement from -3×10^−6^ mm to 3×10^−6^ mm (based on the grayscale image) was used. The vertical and horizontal axes represent the propagation depth (0 cm to 15 cm) and the time after excitation (0.02 s to 0.15 s), respectively. The vibrating source was set at the surface of the liver tissue (i.e., depth of ‘0’ cm). Displacement values acquired before time 0.02 s were excluded from the analysis due to contaminated by longitudinal or compression waves at the onset of the mechanical vibration.

As shown in Fig 2, the peak amplitude of the axial displacements attenuates with the propagation depth. On the subplots, a small variation in grayscale value between superficial and deep tissue regions is indicative of a small attenuation in the peak amplitude of the axial displacement along the depth of propagation, whereas as a large variation in grayscale value is indicative of a sharp attenuation. Therefore, simulations show that a shear wave decays as a function of tissue depth. For a constant viscosity combined with different elasticity (i.e., each column in Fig 2) simulations showed a similar attenuation in the peak amplitude of axial displacement. And the results at a constant elasticity and different viscosity (i.e., each row in Fig 2) show that a higher viscosity significantly increases the shear wave attenuation.

#### Maximum attenuation of the axial displacement as a function of tissue depth

**Fig 3:**
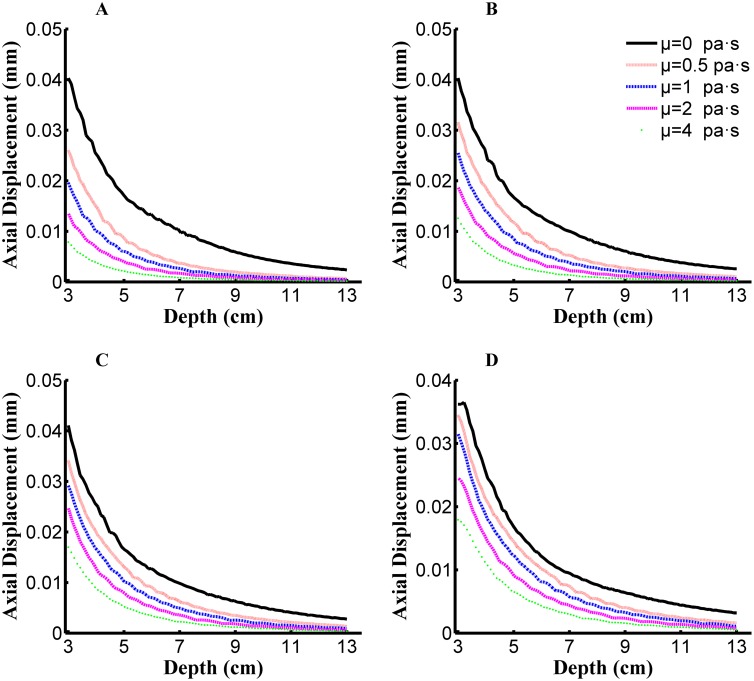
Peak axial displacements along the depth of propagation for the simulated dataset, viscosity (*μ*_2_) are set as 0/0.5/1/2/4 Pa·s in per subfigure. **Young's modulus (E) in each subfigure:** (A) E = 4 kPa, (B) E = 6 kPa, (C) E = 8 kPa, and (D) E = 10 kPa.

Based on simulation results, attenuation in the peak amplitude of axial displacement is influenced by both viscosity and elasticity. Peak axial displacement as a function of propagation depth and viscosity is shown in Fig 3 for different elasticity values. The attenuation of the peak axial displacements with depth can be observed. The peak axial displacement as a function of tissue depth can be approximated by an *n*-order polynomial or exponential function. Coefficients used to fit the function to the data curve are influenced by both tissue elasticity and viscosity.

A quadratic function is used to fit the data curve, with the ‘goodness of fit’ (R^2^ ≥0.99) achieving satisfactory precision for all simulation conditions. The quadratic function was defined as:

$$Axial\;\;displacement=a\times Depth^{2}+b\times Depth+c$$ (4)

where *a*, *b*, and *c* were the fitting parameters.

**Fig 4:**
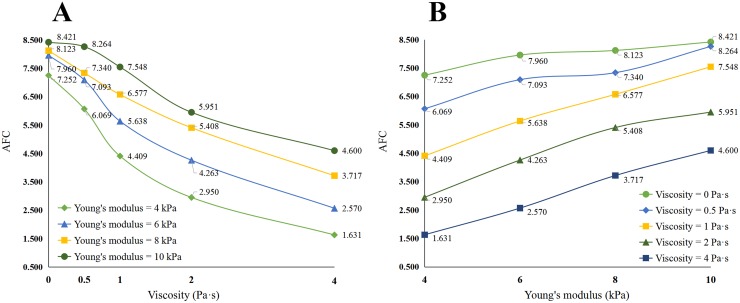
AFC varied with viscosity or Young's modulus. (A) Changing curve with viscosity. (B) Changing curve with Young's modulus.

Curve fitting was completed for each set of 10 TE assessments performed on each patient, yielding 10 sets of parameters for each patient. After excluding exceptional values, using Grubbs criterion and Dixon criterion, fitting parameters were averaged to define one set of fitting parameters for each patient. Among the fitting parameters defined, parameters *b* and *c* are influenced by the location of the quadratic polynomial curve, whereas parameter *a* defined the form of the attenuation curve, namely its morphology and the slope of attenuation (i.e., the AFC value). The simulated AFC for different combinations of elasticity and viscosity is shown in Table 1, with effects of changing viscosity or Young's modulus on the AFT shown in Fig 4.

**Table 1:** AFC values for different combinations of viscosity and Young's modulus.

		Viscosity				
		0 Pa·s	0.5 Pa·s	1 Pa·s	2 Pa·s	4 Pa·s
**Young's modulus**	**4 kPa**	7.252	6.069	4.409	2.950	1.631
	**6 kPa**	7.96	7.093	5.638	4.263	2.570
	**8 kPa**	8.123	7.34	6.577	5.408	3.717
	**10 kPa**	8.421	8.264	7.548	5.951	4.600

As shown in Table 1 and in Fig 4, AFC is influence1d by both viscosity and elasticity. Moreover, a large viscosity increases the sensitivity of the AFC to Young’s modulus, compared to a small viscosity (large viscosity, 1.631~4.600 *versus* low viscosity, 7.252~8.421; Fig 4A). In contrast, the AFC is more sensitive to changes in viscosity when Young’s modulus is small, rather than large (small Young’s modulus, 1.631~7.252 *versus* large Young’s modulus, 4.600~8.421; Fig 4B). Therefore, in early stages of liver fibrosis (i.e., small change in Young’s modulus), the AFC value would reflect changes in liver viscosity.

### Analysis of the clinical data

#### Participants

Among the 108 prospective patients for our study, 9 were excluded for the following reasons: invalid LSM values in 6 patients, either due to a detection success rate < 60% or high interquartile range of values; hepatitis C virus coinfection in 1 patients; and exacerbation of hepatitis infection in 1 patient;pericardial tumor in 1 patient. Relevant characteristics of the remaining 99 patients are summarized as follows: males, 64 (64.60%) and females, 35 (35.40%); median age, 37.7 years. The METAVIR classification scores of liver fibrosis are summarized in Table 2. Our study group included 81 cases of F1 stage (81.80%) and 18 cases of F2 stage (18.20%) of early liver cirrhosis; BMI: 23.87±3.42; LSM: 7.06±1.85; Kpa: ALT: 46.73±30.19U/I; AST: 31.87±18.94U/I; TBIL: 14.11±5.40mmol/l; DBIL: 4.51±1.89mmol/l. Detail information has shown in Table 2.

**Table 2:** General characteristics of the 99 patients included in the study with staging and grading of fibrosis an0064 necro-inflammatory activity, classified using the METAVIR scoring system.

Factor	Clinical data
**Male, ‘n’ (%)**	64 (64.60%)
**Female, ‘n’ (%)**	35 (35.40%)
**Age, years**	37.70±9.97
**BMI**	23.87±3.42
**LSM (kPa)**	7.06±1.85
**F1, ‘n’ (%)**	81 (81.80%)
**F2, ‘n’ (%)**	18 (18.20%)
**ALT(U/I)**	46.73±30.19
**AST(U/I)**	31.87±18.94
**TBIL(mmol/I)**	14.11±5.40
**DBIL(mmol/I)**	4.51±1.89
**Total number**	99

Notes: All the data are reported as statistical quantiles (percentage) or mean±standard deviation, as appropriate for the dataset. LSM: Liver stiffness measurement; BMI: body Mass Index; ALT: alanine transaminase; AST: aspartate aminotransferase; TBIL: total bilirubin; DBIL: direct bilirubin.

#### LSM and AFC

**Fig 5:**
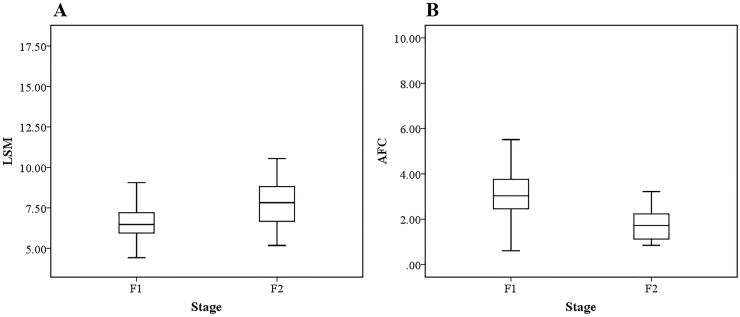
Boxplot of LSM and AFC grouped by two stage of fibrosis. (A) Boxplot of LSM values, and. (B) Boxplot of AFC values.

The LSM values were obtained directly by FibroTouch for each patient, with an overall mean LSM value of 7.06±1.85 kPa (range, 4.42~16.2 kPa). The LSM value for the F1 and F2 subgroups were 6.82±1.70 kPa (range, 4.42~16.20, kPa) and 8.10±2.16 kPa (range, 5.17~14.21 kPa), respectively. Curve fitting of peak displacements, as a function of depth, was performed for each patient to obtain the AFC value. The overall AFC value was 2.92±1.28 (range, 0.61~9.05), with a value of 3.18±1.24 (range, 0.61~9.05) for the F1 group and 1.76±0.71 (range, 0.84~3.22) for the F2 group. Boxplots of the LSM and AFC values, grouped by stage of fibrosis, are shown in Fig 5.

Spearman's correlation analysis identified a positive correlation (P < 0.01) between AFC and LSM values, and a negative correlation (P < 0.001) between the AFC index and stage of liver fibrosis (Table 3). Therefore, although the degree of early-stage liver fibrosis (F1 and F2 stages) correlated significantly with both LSM and AFC, the correlation coefficient was higher for the AFC index (Correlation coefficient = -0.488) than for the LSM value (Correlation coefficient = 0.279). The AFC may provide a more valid index to differentiate F1 and F2 stages of early liver fibrosis. In independent-samples *t*-test (Table 4), the difference between F1 and F2 were both significant for LSM (P < 0.01) and AFC (P < 0.001). The statistical advantage of the AFC over the LSM in differentiating early stages of liver fibrosis was further defined by the *t*-test analysis: AFC, *t*-statistic = 4.652, mean difference = 1.418, standard error of the mean difference = 0.305; *versus* LSM, *t*-statistic = -2.722, mean difference = -1.271, standard error of the mean difference = 0.467.

**Table 3:** Correlation between clinical indicators of liver fibrosis and the LSM and AFC indices.

	LSM		AFC	
	Correlation coefficient	Significance (2-tailed)	Correlation coefficient	Significance (2-tailed)
**Stage**	0.279**	0.005	-0.488***	0.000

** the significance level of the statistics P < 0.01.

*** the significance level of the statistics P < 0.001.

**Table 4:** Independent-samples t-test evaluation of the capacity of the LSM and AFC in distinguishing early stages of liver fibrosis (F1 and F2).

Evaluation indices	LSM	AFC
***t*-statistic**	-2.722**	4.652***
**Significance (2-tailed)**	0.008	0.000
**Mean difference**	-1.271	1.418

Notes: between-group differences evaluated using independent-samples t-test;

** the significance level of the statistics P < 0.01.

*** the significance level of the statistics P < 0.001.

#### Receiver operating characteristic (ROC) analysis

**Fig 6:**
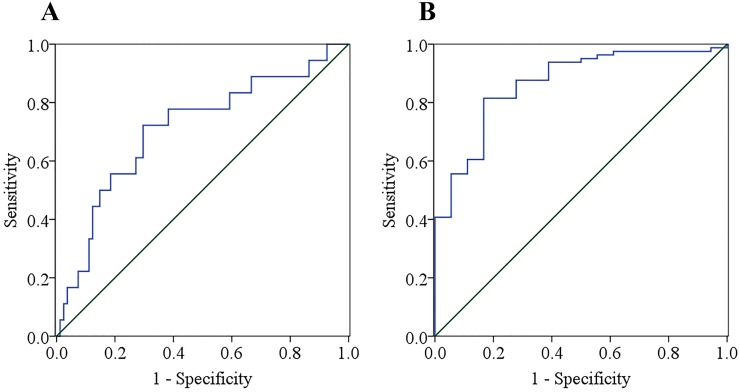
ROC curves for the LSM and AFC indices to differentiate F1 and F2 stages of liver fibrosis. (a) ROC curves of LSM. (b) ROC curves of AFC.

The ROC analysis was conducted to predict liver fibrosis from the combination of LSM and AFC. F1 and F2 stages were determined by liver biopsy, with F1 stage of liver fibrosis defined as a negative result for the ROC analysis, and F2 stage defined as a positive result. The ROC curves are shown in Fig 6. The Youden index, sensitivity, specificity, positive (PPV) and negative (NPV) predictive values, as well as diagnostic accuracy were evaluated for the two indices, with summary statistics reported in Table 5. The diagnostic value of the LSM and AFC indices were compared using the area under the receiver operating characteristic (AUROC), with a value closer to 1 being indicative of a higher diagnostic value for early-stage liver fibrosis.

**Table 5:** Area under the ROC curve and summary statistics of the diagnostic value of the LSM and AFC indices for stages of liver fibrosis ≥ F2.

	LSM	AFC
**AUROC**	0.709**	0.866***
**Significance (2-tailed)**	0.006	0.000
**Cut-off value**	7.042	2.256
**Youden index**	0.426	0.648
**Sensitivity**	72.22%	83.33%
**Specificity**	70.37%	81.48%
**PPV**	35.14%	50.00%
**NPV**	91.94%	95.65%
**Diagnose accuracy**	70.71%	81.82%

** the significance level of the statistics P < 0.01.

*** the significance level of the statistics P < 0.001.

Including all 99 cases, the AUROC for stage of liver fibrosis ≥ F2 was 0.708 for the LSM and 0.866 for the AFC, with the AFC index being superior to LSM in differentiating F1 and F2 stages of early liver fibrosis. The optimal cut-off value was 7.042 for the LSM and 2.256 for the AFC index for stage of liver fibrosis ≥ F2. The Youden index, sensitivity, specificity, PPV, NPV, and diagnostic accuracy of the AFC index (0.648, 83.33%, 81.48%, 50.00%, 95.65%, and 81.82%) were all superior to values for the LSM (0.426, 72.22%, 70.37%, 35.14%, 91.94%, 70.71%), as shown in Table 5.

#### Influential factors of AFC

Correlation results on influential factors of AFC has been given in this section. The analyzed factors included LSM, Age BMI and some blood test results. Statistical analyses were done in clinic data by SPSS, Spearman’s correlation coefficient was calculated between AFC and other indexes. As shown in Table 6.

**Table 6:** Correlation between clinical indicators of liver fibrosis and the AFC indices.

	correlation coefficient	P-value
**LSM**	0.450***	0.000
**Age**	0.046	0.654
**BMI**	-0.019	0.854
**ALT**	0.330**	0.001
**AST**	0.265**	0.008
**TBIL**	0.124	0.221
**DBIL**	0.167	0.098

** the significance level of the statistics P < 0.01.

*** the significance level of the statistics P < 0.001.

Spearman’s correlation analysis show that there were statistically significant correlation between LSM, ALT, AST and shear wave attenuation fitting coefficient (AFC), respectively, correlation coefficient were 0.450 for LSM (P = 0.000), 0.330 for ALT (P = 0.001), as well as 0.265 for AST(P = 0.008). There were not statistically significant correlation between AGE, BMI, TBIL, DBIL and shear wave attenuation fitting coefficient, respectively, correlation coefficient and bilateral significance were 0.046 for AGE, 0.019 for BMI, 0.124 for TBIL, 0.167 for AGE (All P-value>0.05).

#### Diagnostic value for combine LSM with AFC

Stepwise regression analysis was used to select a best group of factors for the prediction of liver fibrosis stages, and the multiple linear regression model established. The new forecasted quantity was defined as liver fibrosis forecast value (LFFV). LFFV which was a new index to evaluate liver fibrosis, was constituted of the linear combination of LSM and AFC. The significance test of regression equation probability by analysis of variance(ANOVA) was less than 0.05 significant level. The regression equation of the LFFV could be decided in following formula:

LFFV=0.847×LSM−0.947×AFC+0.763 (5)

Results indicate: in the whole 99 cases of patients, LSM, AFC and LFFV had AUROCs of 0.709, 0.866 and 0.977 for F2, respectively. There was no significant difference of AUROC between evaluative standards of LSM and AFC but significant improving by the standard of LFFV. The optimal cut-off values of three standards for all disease patients were 7.042, 2.256 and 4.345 for F2. Youden index, sensitivity, specificity, PPV, NPV as well as diagnose accuracy of LFFV (0.889, 100.00%, 89.89%, 66.67%, 100.00%, and 90.91%) has all obvious improvement against LSM and AFC.

## Discussion

Liver disease is prevalent in China and its diagnosis and treatment is a relevant clinical issue. Chronic liver diseases usually develop gradually and are associated with a process of liver fibrosis. As the early stage of liver fibrosis can be reversed with treatment, there has been increasing emphasis placed on developing a diagnostic technique to accurately identify liver disease in its early stages. Although elastography has emerged in recent years as a diagnostic technique for liver disease that is superior to conventional imaging techniques, without the risks associated with biopsy, its application remains limited for reliable identification of early stage liver disease, such as the differentiation of F1 and F2 stages of liver disease. The AFC that we developed in this study, based on vibration amplitude attenuation, provides a more accurate diagnosis of early stage liver fibrosis than LSM.

We used numerical simulations to characterize the effects of different viscoelasticity parameters of shear wave propagation through the liver. Propagation characteristics of the shear wave can be obtained from the axial displacement and used to calculate shear wave attenuation along the propagation depth within a tissue. Tissue viscosity affects the attenuation of peak shear wave amplitude as a function of tissue depth, such that higher liver viscosity produces larger amplitude attenuation. Peak axial displacement as a function of the depth can be described by a second-order polynomial function. The second order coefficient, which determines the morphometry of the curve, is affected by both tissue elasticity and viscosity and can be calculated from the fitted curve. Using this mathematical method, we defined a new index, the AFC, which reflects the viscoelastic characteristics of the liver. Salameh et al. [16] demonstrated that the viscosity properties of the liver can provide early identification of liver fibrosis. Based on our simulation data, the AFC index is sensitive to changes in viscosity, associated with early-stage liver fibrosis (i.e., smaller Young’s modulus). Based on our outcomes, we propose that the AFC index provides new information for the diagnosis of liver disease, with specific value in differentiating early stages of liver fibrosis.

In the clinical phase of the study, we reviewed 108 cases of liver fibrosis resulting from chronic hepatitis B infection, with 99 cases meeting our final selection criteria and included in the analysis. While tissue elasticity can be obtained by using existing technology (FibroScan or FibroTouch), the AFC can be derived from shear wave attenuation information obtained by TE.

Our numerical simulations described an inverse relationship between tissue viscosity and the AFC index: the higher the liver viscosity, the smaller the AFC index. Our correlation analysis identified a further negative correlation between the AFC index and early-stage liver fibrosis (P < 0.01). Based on our simulation results and our clinical tests, we propose that there exists a clinically meaningful relationship between liver viscosity and early-stage liver fibrosis, with our results being comparable to those previously published by other authors [15, 17]. The AFC index was more responsive than the LSM in differentiating F1 and F2 stages of liver fibrosis. Based on our ROC curve analysis, an AFC index <2.256 provided an optimal cut off to identify an F2 stage of liver fibrosis. This AFC cut off provided greater reliability and diagnostic accuracy than the cut off for LSM (>7.042). Youden’s index (0.648), sensitivity (83.33%), specificity (81.48%), PPV (50.00%), NPV (95.65%) and diagnostic accuracy (81.82%) of the AFC were all higher than those of the LSM (0.426, 72.22%, 70.37%, 35.14%, 91.94%, and 70.71%, respectively). Moreover, for the 99 patients included in our case series, the evaluative standard of the AFC index had higher accuracy than the LSM in differentiating F1 from F2 fibrosis (AUROC of 0.708 for AFC vs. 0.866 for LSM). Therefore, the AFC index may provide clinicians with an accurate diagnosis of early-stage liver fibrosis and should be considered as a complementary diagnostic measure of TE. The change in AFC implies that, with the exception of liver elasticity, viscosity may also play an important role in early-stage fibrosis. The AFC index, which combines measurements of viscosity and elasticity to evaluate liver fibrosis, could avoid the need for biopsy procedure in most patients with chronic liver disease resulting from hepatitis B infection. Spearman’s correlation analysis showed that AFC was influenced by the level of LSM, ALT. However, there are no correlation between AFC and Age, BMI, TBIL or DBIL.

LSM and AFC were substituted into regression equation to obtain LFFV as the new evaluative standard. LFFV has all obvious improvement against LSM and AFC in Youden index, sensitivity, specificity, PPV, NPV as well as diagnose accuracy. Evaluative standard of LFFV has been reported to be more accurate than LSM and AFC in differentiating F1 from F2 fibrosis (AUROCs of 0.977 for LFFV vs. 0.866 for AFC and 0.709 for LSM) in 99 cases patients. Moreover, evaluative standard of LFFV has been reported to be more accurate than LSM and AFC in differentiating F1 from F2 fibrosis in 99 cases patients.

The limitations of our study need to be considered in the interpretation of our results. With regards to our numerical modeling, further simulation scenarios need to be evaluated to further extract potentially relevant characteristics, with the feasibility of these characteristics verified against clinical data. Moreover, our clinical findings need to be verified in a larger study aiming to evaluate the responsiveness and reliability of viscosity parameters for the diagnosis of liver disease of varying grades. These further research schemes might provide strong support to liver disease diagnosis for application of viscosity parameter.

## Supporting Information

S1 Fig Finite element model of liver tissue and the mesh generated. (DOCX)

S1 Table Patients’ information data. (XLSX)
