# Supplementary material for: Evaluating the Significance of Viscoelasticity in Diagnosing Early-Stage Liver Fibrosis with Transient Elastography
Source: PLoS One. 2017 Jan 20;12(1):e0170073. doi: 10.1371/journal.pone.0170073 (PMC5249210; doi:10.1371/journal.pone.0170073)
Supplement: S1 Fig — (DOCX) [file pone.0170073.s001.docx]

**S1 Fig .** **Finite element model of liver tissue and the mesh generated.** (A) An idealized 3D model. (B) An idealized two-dimensional axisymmetric finite element model of liver tissue adopted in our study. (C) The generated finite element mesh (22,500 finite elements).


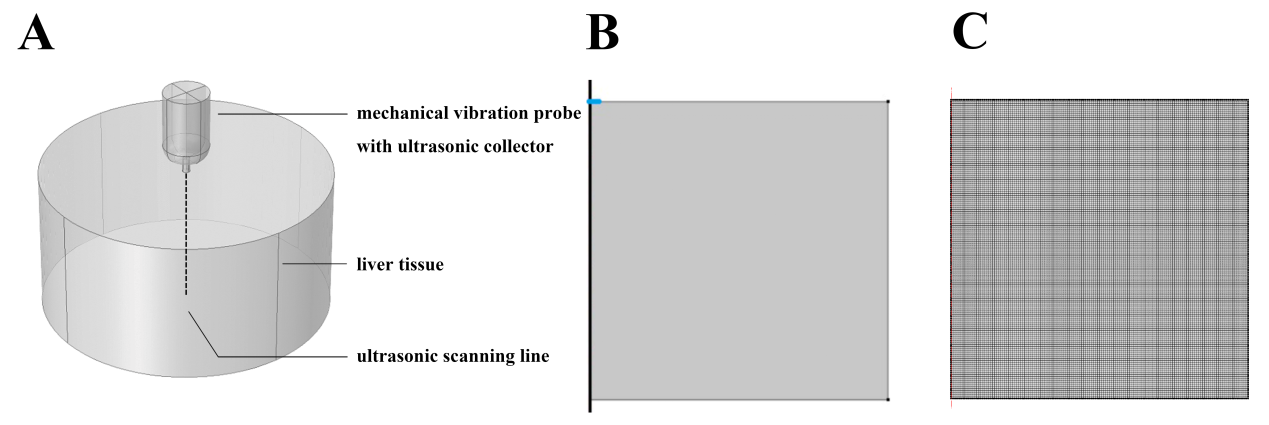


**S2 Fig** **.** **Axial displacement, produced by an external mechanical vibration, measured along the scanning line in the liver tissue model as a function of time (0.02s to 0.15s after excitation). The following combinations of Young's modulus (*E*) and viscosity (*μ*_2_) were used for simulations:** (A) 4 kPa+0 Pa·s, (B) 4 kPa +1 Pa·s, (C) 4 kPa+4 Pa·s, (D) 6 kPa+0 Pa·s, (E) 6 kPa+1 Pa·s, (F) 6 kPa+4 Pa·s, (G) 8 kPa+0 Pa·s, (H) 8 kPa+1 Pa·s, (I) 8 kPa+4 Pa·s, (J) 10 kPa+0 Pa·s, (K) 10 kPa+1 Pa·s and (L) 10 kPa+4 Pa·s, respectively.


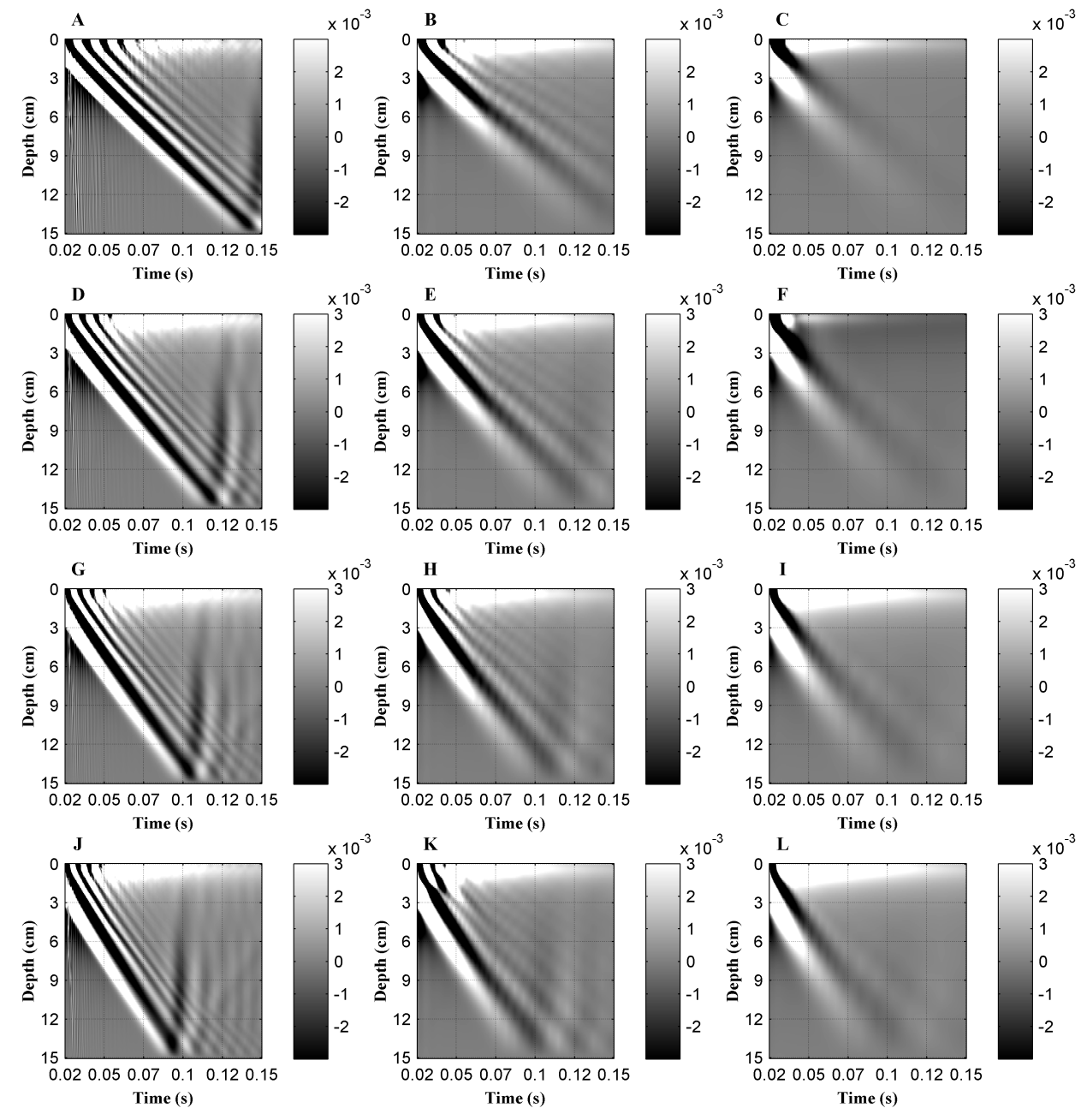


**S3 Fig . Peak axial displacements along the depth of propagation for the simulated dataset, viscosity (*μ*_2_) are set as 0/0.5/1/2/4 Pa·s in per subfigure. Young's modulus (E) in each subfigure:** (A) E = 4 kPa, (B) E = 6 kPa, (C) E = 8 kPa, and (D) E = 10 kPa.


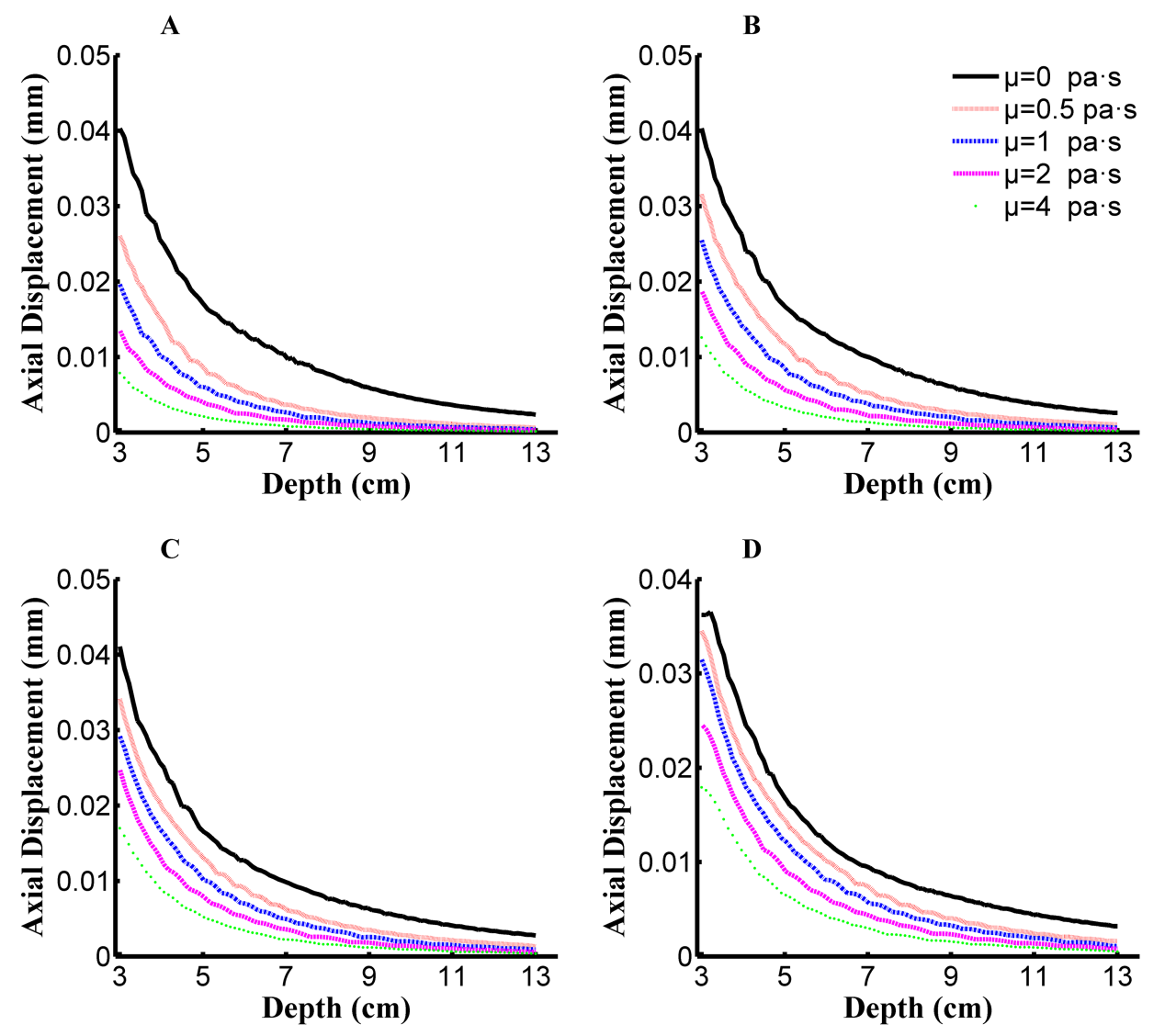


**S4 Fig .** **AFC varied with viscosity or Young's modulus.** (A) Changing curve with viscosity. (B) Changing curve with Young's modulus.


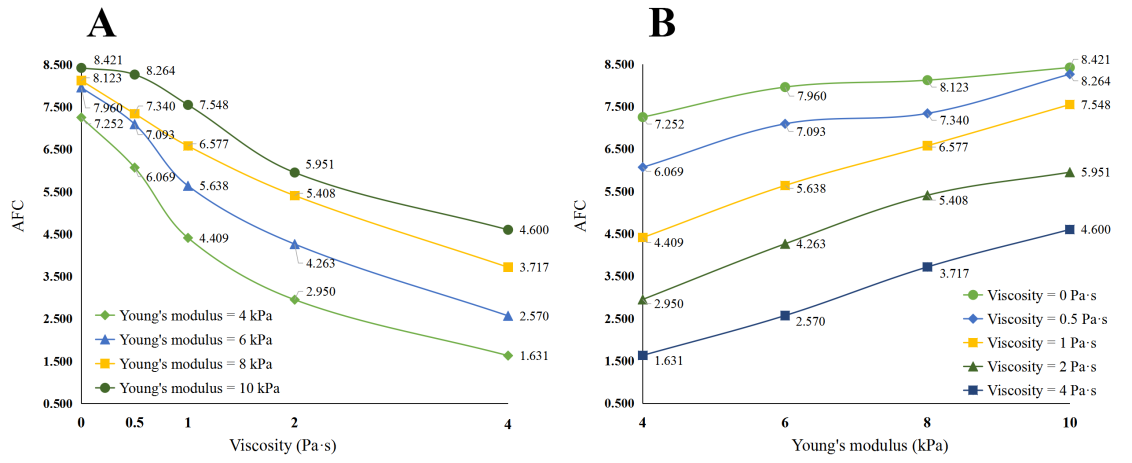


**S5 Fig. Boxplot of LSM and AFC grouped by two stage of fibrosis.** (A) Boxplot of LSM values, and. (B) Boxplot of AFC values.


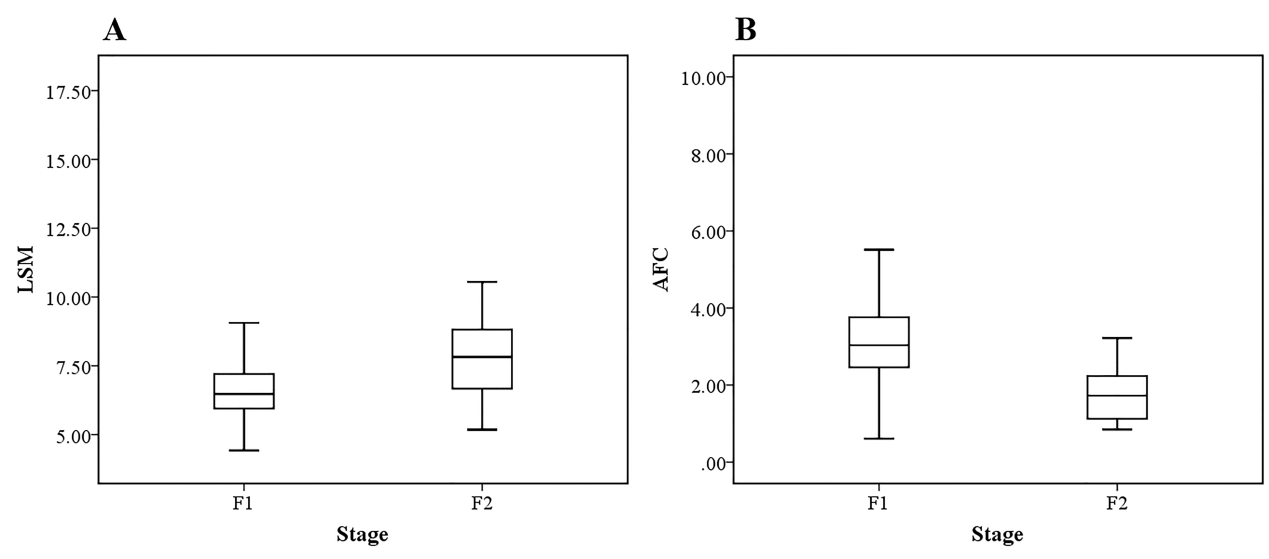


**S6 Fig . ROC curves for the LSM and AFC indices to differentiate F1 and F2 stages of liver fibrosis.** (a) ROC curves of LSM. (b) ROC curves of AFC.

**
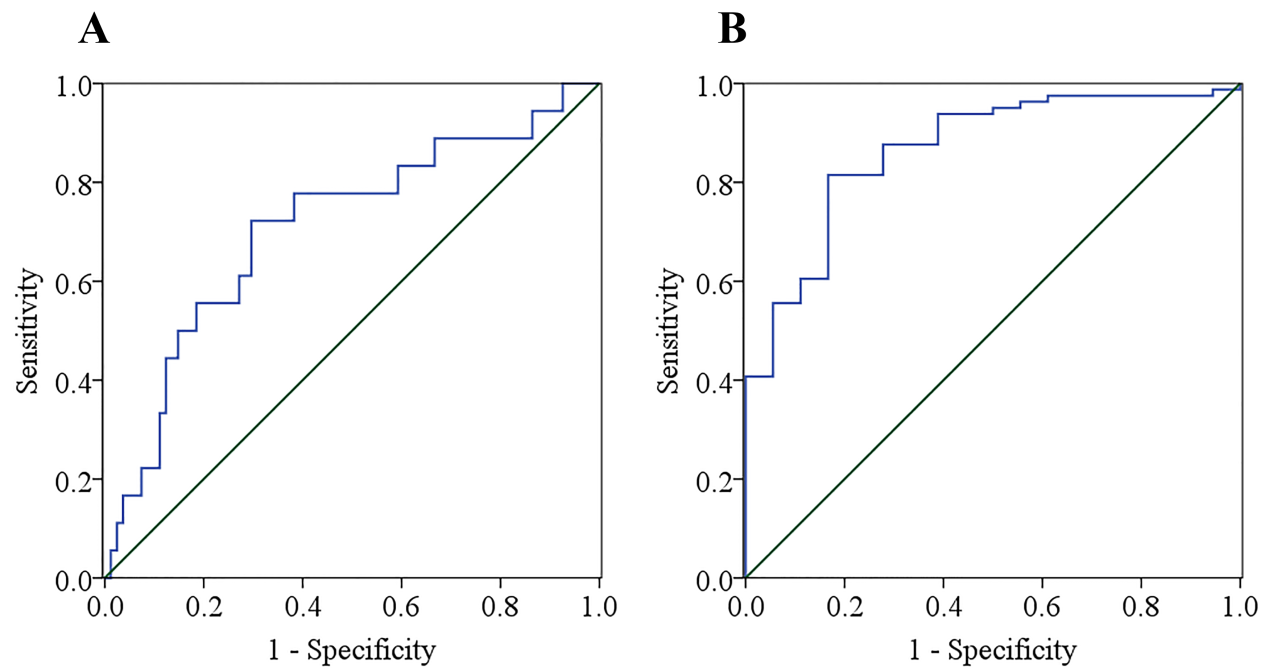
**

**S1 Table . AFC values for different combinations of viscosity and Young's modulus**

|  |  | **Viscosity** | | | | |
| --- | --- | --- | --- | --- | --- | --- |
|  |  | **0 Pa·s** | **0.5 Pa·s** | **1 Pa·s** | **2 Pa·s** | **4 Pa·s** |
| **Young's modulus** | **4 kPa** | 7.252 | 6.069 | 4.409 | 2.950 | 1.631 |
|  | **6 kPa** | 7.96 | 7.093 | 5.638 | 4.263 | 2.570 |
|  | **8 kPa** | 8.123 | 7.34 | 6.577 | 5.408 | 3.717 |
|  | **10 kPa** | 8.421 | 8.264 | 7.548 | 5.951 | 4.600 |

**S2 Table . General characteristics of the 99 patients included in the study with staging and grading of fibrosis and necro-inflammatory activity, classified using the METAVIR scoring system.**

| **Factor** | **Clinical data** |
| --- | --- |
| **Male, ‘n’ (%)** | 64 (64.60%) |
| **Female, ‘n’ (%)** | 35 (35.40%) |
| **Age, years** | 37.70±9.97 |
| **BMI** | 23.87±3.42 |
| **LSM (kPa)** | 7.06±1.85 |
| **F1, ‘n’ (%)** | 81 (81.80%) |
| **F2, ‘n’ (%)** | 18 (18.20%) |
| **ALT（U/I）** | 46.73±30.19 |
| **AST(U/I)** | 31.87±18.94 |
| **TBIL(mmol/I)** | 14.11±5.40 |
| **DBIL(mmol/I)** | 4.51±1.89 |
| **Total number** | 99 |

**S3 Table . Correlation between clinical indicators of liver fibrosis and the LSM and AFC** **indices.**

|  | **LSM** | | **AFC** | |
| --- | --- | --- | --- | --- |
|  | **Correlation coefficient** | **Significance (2-tailed)** | **Correlation coefficient** | **Significance (2-tailed)** |
| **Stage** | 0.279** | 0.005 | -0.488*** | 0.000 |

** the significance level of the statistics P < 0.01.

*** the significance level of the statistics P < 0.001.

**S4 Table . Independent-samples t-test evaluation of the capacity of the LSM and AFC in distinguishing early stages of liver fibrosis (F1 and F2).**

| **Evaluation indices** | **LSM** | **AFC** |
| --- | --- | --- |
| ***t*-statistic** | -2.722^**^ | 4.652^***^ |
| **Significance (2-tailed)** | 0.008 | 0.000 |
| **Mean difference** | -1.271 | 1.418 |

Notes: between-group differences evaluated using independent-samples t-test;

** the significance level of the statistics P < 0.01.

*** the significance level of the statistics P < 0.001.

**S5 Table . Area under the ROC curve and summary statistics of the diagnostic value of the LSM and AFC indices for stages of liver fibrosis ≥ F2.**

|  | **LSM** | **AFC** |
| --- | --- | --- |
| **AUROC** | 0.709** | 0.866*** |
| **Significance (2-tailed)** | 0.006 | 0.000 |
| **Cut-off value** | 7.042 | 2.256 |
| **Youden index** | 0.426 | 0.648 |
| **Sensitivity** | 72.22% | 83.33% |
| **Specificity** | 70.37% | 81.48% |
| **PPV** | 35.14% | 50.00% |
| **NPV** | 91.94% | 95.65% |
| **Diagnose accuracy** | 70.71% | 81.82% |

**S6 Table . Correlation between clinical indicators of liver fibrosis and the AFC indices.**

|  | **correlation coefficient** | **P-value** |
| --- | --- | --- |
| **LSM** | 0.450^***^ | 0.000 |
| **Age** | 0.046 | 0.654 |
| **BMI** | -0.019 | 0.854 |
| **ALT** | 0.330^**^ | 0.001 |
| **AST** | 0.265^**^ | 0.008 |
| **TBIL** | 0.124 | 0.221 |
| **DBIL** | 0.167 | 0.098 |

** the significance level of the statistics P < 0.01.

*** the significance level of the statistics P < 0.001.
